# Neonatal and neurodevelopmental outcomes in preterm infants according to maternal body mass index: A prospective cohort study

**DOI:** 10.1371/journal.pone.0225027

**Published:** 2019-12-05

**Authors:** Marie Moreau, Mathilde Remy, Simon Nusinovici, Valérie Rouger, Lisa Molines, Cyril Flamant, Guillaume Legendre, Jean-Christophe Roze, Agnès Salle, Patrick Van Bogaert, Régis Coutant, Géraldine Gascoin

**Affiliations:** 1 Department of Neonatal Medicine, Angers University Hospital, Angers, France; 2 CIC 1413, Nantes University Hospital, Nantes, France; 3 Loire Infant Follow-Up Team (LIFT) Network, Pays de Loire, France; 4 Department of Neonatal Medicine, Nantes University Hospital, Nantes, France; 5 Department of Obstetrics and Gynaecology, Angers University Hospital, Angers, France; 6 Department of Endocrinology, Diabetology and Nutrition, Angers University Hospital, Angers, France; 7 Department of Paediatric Neurology, Angers University Hospital, Angers, France; 8 Department of Paediatric Endocrinology, Angers University Hospital, Angers, France; Amsterdam Universitair Medische Centra, NETHERLANDS

## Abstract

**Objective:**

Maternal obesity is associated with an increase in maternal, foetal and neonatal morbidity and mortality. The aim of our study was to evaluate the relationships between maternal pre-pregnancy body mass index and (1) neonatal outcome in preterm infants, and (2) neurodevelopmental outcome at 2 years of corrected age.

**Method:**

We conducted a single-centre cohort study. Infants born between 24+0 and 33+6 weeks of gestation between January 2009 and December 2013, hospitalised in the neonatal intensive care unit of Angers University Hospital, and with available data regarding maternal pre-pregnancy body mass index were eligible. Three groups were defined according to maternal body mass index: normal (n = 418), overweight (n = 136) and obese (n = 89). The primary outcome was neurodevelopment at 2 years of corrected age. Children with a non-optimal neuromotor and/or psychomotor assessment and/or a sensory disability were regarded as having a “non-optimal neurodevelopmental outcome”. Neuromotor function was regarded as non-optimal when cerebral palsy was present or when the clinical examination revealed neurological signs of abnormal muscular tone. Psychomotor assessment was regarded as non-optimal if the revised Brunet-Lézine test was < 85 or when the overall score in the parental Ages and Stages Questionnaire (ASQ) was < 185. Finally, sensory disabilities such as blindness and children who required a hearing aid were taken into account. The secondary outcome was the composite criteria of neonatal complications. Multivariable analysis included the following variables: mother’s age, gestational age, smoking during pregnancy, magnesium sulphate and steroid treatment during pregnancy, twin status, gender, socioeconomic status and social security benefits for those with low incomes.

**Results:**

The study population was composed of 643 preterm infants. Among them, 520 were assessed at 2 years. There was no difference in the proportion of infants with non-optimal neurodevelopmental outcomes between the three groups (16.6% for obese, 13.5% for overweight, 16.9% for normal body mass index mothers; p = 0.73). According to multivariable analysis, being born from an overweight or obese mother was not associated with an increased risk of non-optimal neuro-development at 2 years (adjusted OR = 0.84 [0.40–1.76] for obese, adjusted OR = 0.83 [0.43–1.59] for overweight mothers). There was no difference in the proportion of preterm infants with a non-optimal composite criterion of neonatal complications between the three groups. In the multivariable analysis, being born from an overweight or obese mother was not associated with an increased risk of non-optimal neonatal outcomes (adjusted OR = 0.95 [0.49–1.83] for obese, adjusted OR = 1.18 [0.69–2.01] for overweight mothers).

**Conclusion:**

In this large prospective cohort of preterm infants born before 34 weeks of gestation, we found no relationship between maternal body mass index and neurodevelopmental outcomes at 2 years of corrected age and no relationship between maternal body mass index and neonatal outcomes. Other prematurity-related factors may be more relevant for neurodevelopmental outcome than the mother’s pre-pregnancy BMI.

## Introduction

Obesity is a major public health concern, with its prevalence having increased over the last 30 years [1]. Almost 25% of pregnant women in Europe and almost 50% of pregnant women in the United States attending their first prenatal consultation are either overweight or obese [1,2].

Maternal obesity is associated with an increase in maternal, foetal and neonatal morbidity and mortality which is proportional to pre-conceptional body mass index (BMI) [3,4]. Indeed, maternal adiposity increases the risk of gestational diabetes, pre-eclampsia, pregnancy-induced hypertension, instrumental and caesarean delivery, preterm birth, large-for-gestational-age newborns, foetal defects, congenital anomalies, and perinatal death [5–12].

Moreover, maternal obesity is associated with an increase in systemic and brain inflammation [13]. Maternal obesity also increases the risk of cerebral palsy for full-term neonates [14] and the risk of impaired offspring development among extreme preterm infants born before 28 weeks of gestation [15]. It was also associated with a positive screening for autism and a lower composite language score at the age of 2 in a cohort of preterm infants born before 30 weeks of gestation [15,16].

The aim of our study was to analyse the relationship between maternal pre-pregnancy BMI (1) and neonatal outcomes (2) and neurodevelopmental outcomes at 2 years of corrected age, for preterm infants born before 34 weeks of gestation.

## Materials and methods

### Study population

We conducted a single-centre cohort study with secondary analysis of a prospective study. All infants born between 24+0 and 33+6 weeks of gestation, between January 2009 and December 2013 and hospitalised in the neonatal intensive care unit (NICU) of Angers University Hospital, were eligible. Infants with missing data regarding pre-pregnancy maternal BMI were excluded, as were infants who exhibited genetic abnormalities or malformations. Clinical data (obstetrical and neonatal) were collected prospectively for all preterm infants enrolled in the Loire Infant Follow-up Team (LIFT) network [17]. The LIFT network is a prospective multicentric cohort of preterm infants born with a gestational age of 34 weeks or less. Birth weights were expressed in relation to gestational age as z-scores for standard deviations (SD) from Olsen growth curves [18]. Intra-uterine growth restriction (IUGR) was defined by a reduction of foetal growth during gestation and with a birth weight below the 10th percentile. Three groups were defined: overweight mothers with a pre-pregnancy BMI ≥25kg/m^2^ and <30 kg/m^2^, obese mothers with a pre-pregnancy BMI ≥30 kg/m2, and mothers with a normal pre-pregnancy BMI, i.e. 18 to 25 kg/m^2^. Infants born from a mother with a pre-pregnancy BMI <18 kg/m^2^ were excluded. Weight and height before pregnancy were self-reported by mothers at their first prenatal visit and collected retrospectively from the medical records. All maternal BMI were self-reported pre-pregnancy BMI. Cesarean delivery included elective and non-elective one.

### Methods

The primary outcome was neurodevelopment at 2 years of corrected age. Infants were evaluated at two years +/- 2 months of corrected age with routine validated tests [17]. Assessments to define optimal and non-optimal neurodevelopmental outcomes included a physical examination by a LIFT-trained paediatrician specialised in the early detection of neurodevelopmental disorders, a psychomotor evaluation by a LIFT network psychologist, and a parent-completed questionnaire (Ages and Stages Questionnaire, ASQ). Neuromotor evaluation was regarded as non-optimal if infants were unable to walk without aid (cerebral palsy) or if the clinical examination revealed abnormal neurological signs (phasic stretch in the triceps sural muscle and imbalance of passive axial tone with predominance of extensor tone) during independent walking by a corrected age of 2 years (phasic stretch in the triceps sural muscle and imbalance of passive axial tone with predominance of extensor tone) according to the Amiel-Tison criteria [19,20]). Psychomotor evaluation was assessed using the revised Brunet-Lézine test [21]. The development of the initial Brunet-Lezine test and its revision followed rigorous methods, including the evaluation of test-retest reliability and internal reliability, both of which were high. The minimum duration of the test is 30 minutes. It is designed to allow 4 developmental age subscores to be calculated for children who are aged between 2 and 30 months. The revised Brunet-Lezine test covers 4 domains (movement and posture, language, socialisation, coordination) and allows the calculation of 4 subscores which, when combined, yield a global Developmental Quotient (DQ) score. The mean and maximal global scores were 100 and 140, whereby DQ values ≤85 define neurodevelopmental impairment. Infants who were not able to perform a DQ test because their neurologic impairment was too severe were included in the subgroup “DQ ≤85 or DQ not realisable”. Paediatric psychologists were blind to parental socio-economic status and maternal education.

Furthermore, neurodevelopmental outcome was assessed using the parent-completed ASQ [20,22,23]. The questionnaire consists of 30 developmental items to assess five domains of child development: communication, gross motor, fine motor, problem solving and personal-social. For each item, the parents indicate “yes” (10 points), “sometimes” (5 points) or “not yet” (0 points) to represent their child's ability to perform a task. Each domain score was obtained by the sum of the items, compared with established cut-off screening points, and was considered abnormal if the score was 2 SD below the mean. The global ASQ was regarded as abnormal if one domain failed. The total sum of the five scores was also calculated. The maximal overall ASQ score is 300 and a score <185 was considered non-optimal [24]. Finally, sensory disabilities such as blindness and children who required a hearing aid were taken into account. Overall, infants with a non-optimal neuromotor and/or psychomotor assessment and/or a sensory disability were regarded as having a “non-optimal neurodevelopmental outcome.” Infants without a documented physical examination or psychomotor assessment were considered as non-assessable at two years, except for infants with severe neurological disabilities considered as non-optimal.

The secondary outcome was a self-defined composite criteria of major neonatal complications which include at least one (one or more, all weighted the same) of the following complications: Apgar score <7 at 5 minutes, severe neurological injury (severe intraventricular haemorrhage grade III or IV, ventriculomegaly, periventricular leukomalacia, deep nuclear grey matter injury), duration of respiratory assistance (mechanical ventilation and non-invasive ventilation support), bronchopulmonary dysplasia defined by oxygen therapy after 36 weeks of gestational age, patent ductus arteriosus with medical and/or surgical treatment, ulcero-necrotising enterocolitis with a score >2 according to Bell’s Staging Criteria, death before discharge.

### Statistical analyses

Maternal BMI was considered as a three-class variable to investigate the effects of being overweight (25 to 30kg/m^2^) and obesity (>30kg/m^2^) on neonatal and 2-year outcomes, compared to normal weight (18 to 25kg/m^2^). First, the proportion of deceased infants, infants with neonatal complications, and those with non-optimal neurodevelopmental outcome at two years according to the class of maternal BMI were compared using Pearson’s chi-squared test. Then, the relationships between maternal BMI and neonatal outcome and 2-years outcome were estimated using logistic regression. To account for possible confounding factors, the following adjustment variables were considered: the mother’s age (16 to 24, 25 to 37, and 38 to 48 years old), gestational age (32 to 34, 28 to 31, 24 to 27 weeks of gestational age), smoking during pregnancy, magnesium sulphate and steroid treatment during pregnancy, twin status, gender, socioeconomic status and social security benefits for those with low incomes. In order to investigate the existence of possible confounders, both unadjusted and adjusted results were presented. Results were expressed as odds ratios (OR) with 95% confidence intervals (95% CI). A p value < or = 0.05 was considered significant. Analyses were performed using R software.

### Ethical statements

Written informed consent was obtained from all parents before including infants in the LIFT cohort and before gathering the relevant neonatal data from the clinical records. The cohort was registered at the French CNIL (*Commission Nationale de l’Informatique et des Libertés* no. 851117, the ethics committee for the collection of clinical data from patient records). Specific approval to use the data in this study was obtained from the Ethics Committee of Angers University Hospital.

## Results

### Population

**Fig 1:**
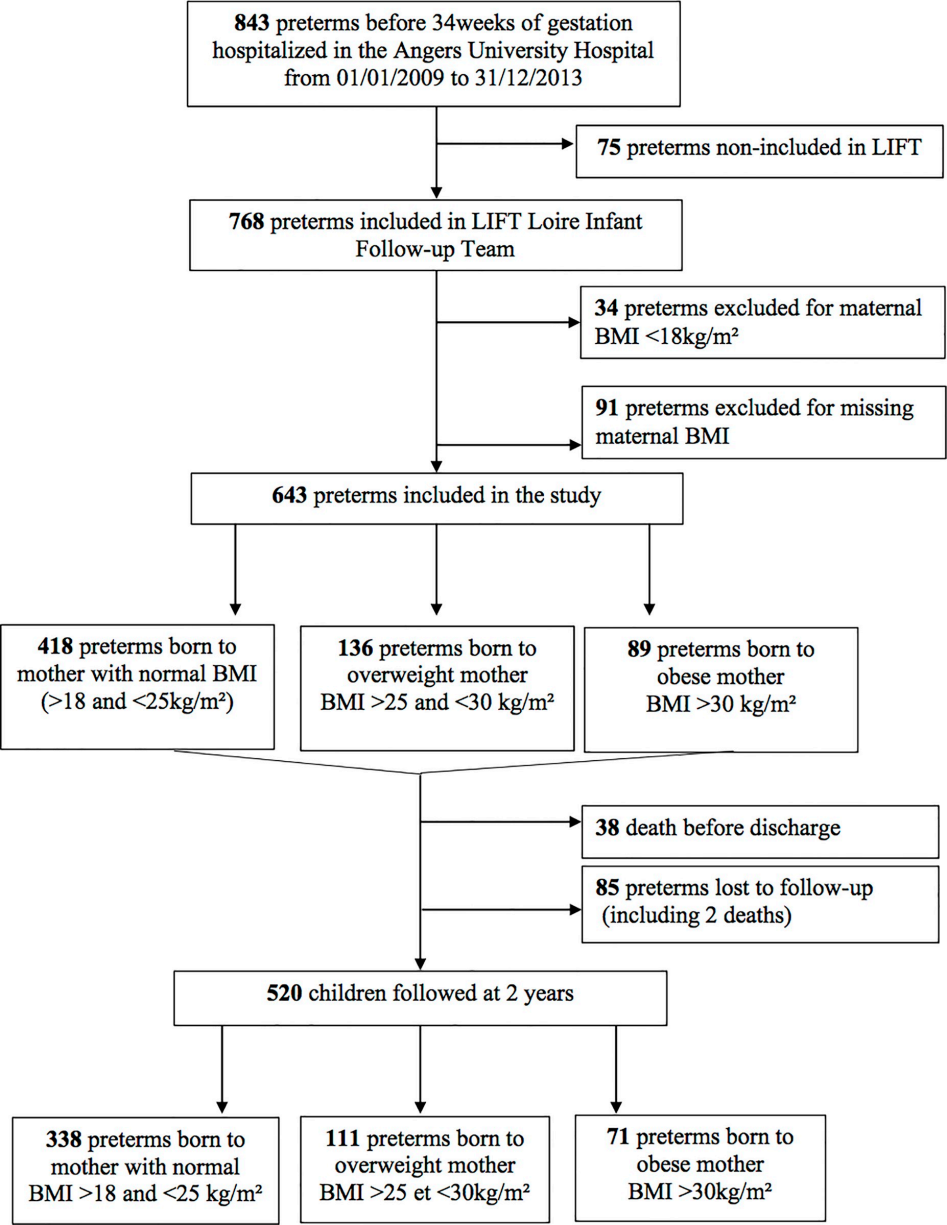
Flow chart.

From January 2009 to December 2013, 843 infants born alive before 34 weeks of gestation were hospitalised in the neonatal intensive care unit of Angers University Hospital (Fig 1). Among them, 75 were not enrolled in the LIFT cohort (due to parental refusal or parents living outside of the region), 34 were excluded because maternal pre-pregnancy BMI was <18kg/m^2^, and 91 were excluded due to missing maternal or neonatal data. Ultimately, the study population was composed of 643 preterm infants. Among them, 520 were assessed at 2 years: 65% (n = 338) were born from mothers with a normal BMI, 21% (n = 111) from overweight mothers, and 14% (n = 71) from obese mothers.

Maternal and neonatal characteristics according to maternal BMI for the overall cohort are presented in Table 1 and Table 2, respectively. Obese mothers required significantly more C-sections, suffered from higher rates of pre-eclampsia and HELLP syndrome and presented more intra-uterine growth restricted foetuses. Moreover, overweight and obese mothers suffered significantly more often from pre-gestational and gestational diabetes. In contrast, obese mothers suffered fewer incidents of preterm labour before 34 weeks of gestational age. Among the overall population, only 3 mothers (0.46%) underwent bariatric surgery. Overweight mothers smoked less during pregnancy. Maternal and neonatal characteristics according to available neurological assessment at 2 years of age are presented in S1 Table. Patients non-assessed at 2 years of corrected age were preterm infants non-included in the study (n = 200), preterm infants dead before discharge (n = 38) and preterm infants lost to follow-up (n = 85) (Fig 1). Preterm infant’s dead before discharge were mostly extremely preterm (24/38, 63%), 76% of them had antenatal steroids and 29% antenatal magnesium sulphate treatment.

**Table 1:** Maternal characteristics.

Maternal BMI (kg/m^2^)	18–25	25–30	>30	p value
	n = 418	n = 136	n = 89	
Maternal age (years)				0.33
16–24	68 (16.3)	24 (17.6)	15 (16.9)	
25–37	314 (75.1)	97 (71.3)	60 (67.4)	
38–48	36 (8.6)	15 (11)	14 (15.7)	
Miscarriages > or = 3	16 (3.8)	7 (5.1)	8 (9)#	0.12
Primiparous	155 (37.1)	41 (30.1)	29 (32.6)	0.30
Social security benefits for individuals with low incomes	62 (14.8)	14 (10.3)	13 (14.6)	0.40
Socio-economic status				0.21
Intermediate	340 (81.3)	115 (84.6)	79 (88.8)	
High	78 (18.7)	21 (15.4)	10 (11.2)	
Smoking during pregnancy	89 (21.3)	15 (11)*	18 (20.2)	0.03
Malformative pathology	18 (4.3)	5 (3.7)	1 (1.1)	0.36
Pregnancy induced hypertension	6 (1.4)	3 (2.2)	5 (5.6)#	0.05
Pre-eclampsia / HELLP syndrome	87 (20.8)	26 (19.1)	34 (38.2)#^	<0.01
Pre-gestational diabetes	1 (0.2)	2 (1.5)	4 (4.5)#	<0.01
Gestational diabetes	17 (4.1)	26 (19.1)*	17 (19.1)#	<0.01
Chorioamnionitis	13 (3.1)	4 (2.9)	2 (2.2)	1
Intra uterine growth restriction	47 (11.2)	12 (8.8)	18 (20.2)#^	0.03
Premature rupture of membranes	62 (14.8)	26 (19.1)	14 (15.7)	0.49
Preterm labour	207 (49.5)	64 (47.1)	29 (32.6)#^	0.01
Medically assisted procreation	72 (17.2)	34 (25)*	19 (21.3)	0.12
Caesarean	260 (62.2)	76 (55.9)	73 (82)#^	<0.01
Magnesium sulphate	105 (25.1)	41 (30.1)	27 (30.3)	0.38
Antenatal steroids	276 (66)	101 (74.3)	62 (69.7)	0.20

number (%). (BMI Body Mass Index; IUGR Intra-Uterine Growth Restriction)

* p-value<0.05; overweight versus normal weight

# p-value<0.05; obese versus normal weight

^ p-value<0.05; obese versus normal overweight

**Table 2:** Neonatal characteristics and neurological outcome at 2 years according to maternal BMI.

Maternal BMI (kg/m^2^)	18–25	25–30	>30	p value
	n = 418	n = 136	n = 89	
Gestational age n (%)				0.32
32–34	162 (38.8)	64 (47.1)	41 (46.1)	
28–31	195 (46.7)	52 (38.2)*	34 (38.2)	
24–27	61 (14.6)	20 (14.7)	14 (15.7)	
Gender n (%)				0.83
Female	184 (44)	64 (47.1)	40 (44.9)	
Male	234 (56)	72 (52.9)	49 (55.1)	
Twin status n (%)	141 (33.7)	51 (37.5)	29 (32.6)	0.67
Birth weight Z-score n (%)				<0.01
<-1 SD	87 (20.9)	23 (17.3)	32 (36)	
-1–0 SD	143 (34.4)	48 (36.1)	33 (37.1)	
0 –+1SD	138 (33.2)	43 (32.3)	20 (22.5) #	
>+1SD	48 (11.5)	19 (14.3)	4 (4.5)	
Delta Z-score birth weight/weight at term mean (SD)	-0.81 (0.94)	-0.69 (0.82)	-0.48 (0.82)#	0.02
Delta Z-score birth weight / weight at 2 years mean (SD)	-0.15 (1.06)	-0.13 (1.11)	0.17 (1.16)#	0.10
Optimal outcome at 2 years old n (%)	282 (83.4)	96 (86.5)	59 (83.1)	0.73
Non-optimal outcome at 2 years old n (%)	56 (16.6)	15 (13.5)	12 (16.9)	

(BMI Body Mass Index; SD Standard Deviation). Z-score: Birth weights were expressed in relation to gestational age as z-scores for standard deviations (SD) from Olsen growth curves. Delta Z-score: Growth during neonatal hospitalisation was assessed by the change in weight z-score between birth and discharge

* p-value<0.05; overweight versus normal weight

# p-value<0.05; obese versus normal weight

^ p-value<0.05; obese versus normal overweight

### Neurodevelopmental outcome at 2 years of corrected age

Regarding the primary outcome, there was no difference in the proportion of infants with non-optimal neurodevelopmental outcome between the three groups (16.6% in the normal BMI group, 13.5% in the overweight group, 16.9% in the obese group, p = 0.73) (Table 2). According to multivariable analysis, being born from an overweight or obese mother was not associated with an increased risk of non-optimal neuro-development at 2 years (adjusted OR = 0.84, [0.40–1.76] for obese mothers, adjusted OR = 0.83, [0.43–1.59] for overweight mothers) (Table 3). Furthermore, male gender was associated with an increased risk of non-optimal neurodevelopment at 2 years. In contrast, having received antenatal steroids was associated with a decreased risk of non-optimal neurodevelopment at 2 years.

**Table 3:** Multivariable analysis: Risk of non-optimal neurodevelopment at 2 years adjusted on gestational age.

	Crude OR	p-value	Adjusted OR	p-value
	(95% CI)		(95% CI)	(Wald’s test)
Maternal BMI (kg/m^2^)				
Reference 18–25	1		1	
25–30	0.79 (0.43–1.46)	0.45	0.83 (0.43–1.59)	0.56
>30	1.02 (0.52–2.03)	0.95	0.84 (0.40–1.76)	0.65
Gestational age (weeks)				
Reference 32–34	1		1	
28–31	1.31 (0.76–2.26)	0.33	1.29 (0.73–2.28)	0.38
24–27	3.27 (1.69–6.35)	<0.01	2.76 (1.37–5.55)	<0.01
Maternal age (years)				
Reference 16–24	1		1	
25–37	1.24 (0.62–2.47)	0.55	1.47 (0.70–3.07)	0.31
38–48	1.90 (0.77–4.67)	0.16	2.51 (0.94–6.69)	0.07
Smoking during pregnancy	1.21 (0.66–2.21)	0.53	0.99 (0.52–1.89)	0.99
Magnesium sulphate	1.49 (0.90–2.45)	0.12	1.29 (0.74–2.22)	0.37
Antenatal corticosteroids	0.56 (0.34–0.90)	0.02	0.55 (0.33–0.91)	0.02
Twins	0.81 (0.49–1.33)	0.40	0.76 (0.45–1.29)	0.32
Male	1.94 (1.18–3.18)	<0.01	2.07 (1.23–3.46)	<0.01
Social security benefit for low incomes	1.47 (0.80–2.71)	0.21	1.59 (0.83–3.03)	0.16
High socio-economic level	0.51 (0.26–1.03)	0.06	0.48 (0.23–1.02)	0.05

(OR Odd Ratio; CI Confidence Interval; BMI Body Mass Index)

### Neonatal outcome

Regarding the secondary outcome, there was no significant difference between the groups with regard to death before discharge, Apgar score at 5 minutes, severe neurological injury, length of respiratory assistance, bronchopulmonary dysplasia at 36 weeks of gestational corrected age, persistent ductus arteriosus that required treatment, and ulcero-necrotising enterocolitis with a score > 2 according to Bell’s Staging Criteria (Table 4). There was no difference in the proportion of preterm infants with non-optimal composite criteria of neonatal complications between the three groups: 28% for preterm infants born from a mother with a normal BMI, 27.2% for preterm infants born from an overweight mother, and 25.8% for preterm infants born from an obese mother (p = 0.92) (Table 4). In the multivariable analysis, being born from an overweight or obese mother was not associated with an increased risk of non-optimal neonatal outcome (adjusted OR = 1.18, [0.69–2.01] for overweight mothers, adjusted OR = 0.95, [0.49–1.83] for obese mothers) (Table 5). Only antenatal corticosteroid administration (one injection or more) was significantly associated with a positive effect on the neonatal outcome. We observed no influence of maternal age, smoking status, twins, gender, or socioeconomic status.

**Table 4:** Neonatal outcome and composite criteria according to maternal BMI.

Maternal BMI (kg/m^2^)	18–25	25–30	>30	p value
	n = 418	n = 136	n = 89	
Death before discharge*	23 (5.5)	8 (5.9)	7 (7.9)	0.69
Apgar < 7 at 5 minutes*	38 (9.5)	10 (8.1)	6 (7.1)	0.73
Severe neurologic injury*	18 (4.3)	10 (7.4)	1 (1.1)	0.08
Length of respiratory assistance**	14 [5–36.2]	10 [4–24]	11 [1–36]	0.11
Broncho-pulmonary dysplasia*				0.72
No oxygen	295 (70.6)	94 (69.1)	67 (75.3)	
< 28 days	95 (22.7)	36 (26.5)	17 (19.1)	
28 days– 36 gestational corrected age	23 (5.5)	5 (3.7)	3 (3.4)	
>36 weeks of gestational corrected age	5 (1.2)	1 (0.7)	2 (2.2)	
Persistent ductus arteriosus with treatment necessary*	75 (17.9)	23 (16.9)	15 (16.9)	0.95
Ulcero-necrotising enterocolitis (Bell’s staging criteria >2)*	13 (3.1)	4 (2.9)	2 (2.2)	1
Optimal neonatal outcome / Composite criteria = 0 *	301 (72)	99 (72.8)	66 (74.2)	0.92
Non-optimal neonatal outcome Composite criteria = or > 1 *	117 (28)	37 (27.2)	23 (25.8)	

*number (%)

**median (interquartile range)

(BMI Body Mass Index; Severe neurologic injury: intraventricular haemorrhage grade III or IV, ventriculomegaly, periventricular leukomalacia, deep nuclear grey matter injury). Composite criteria; one point for each item: death before discharge, Apgar < 7 at 5 minutes, severe neurologic injury, length of respiratory assistance, bronco-pulmonary dysplasia, persistent ductus arteriosus with treatment, ulcero-necrotising enterocolitis with Bell-s staging criteria >2).

**Table 5:** Multivariable analysis: risk of non-optimal neonatal outcome adjusted on gestational age.

	Crude OR	p-value	Adjusted OR	p-value
	(95% CI)		(95% CI)	(Wald’s test)
Maternal BMI (kg/m^2^)				
Reference 18–25	1		1	
25–30	0.96 (0.62–1.48)	0.86	1.18 (0.69–2.01)	0.55
>30	0.90 (0.53–1.51)	0.68	0.95 (0.49–1.83)	0.88
Gestational age (weeks)				
Reference 32–34	1		1	
28–31	3.93 (2.38–6.5)	<0.01	3.87 (2.30–6.52)	<0.01
24–27	48.68 (24.74–95.77)	<0.01	49.38 (24.37–100.04)	<0.01
Maternal age (years)				
Reference 16–24	1		1	
25–37	1.09 (0.68–1.75)	0.71	0.96 (0.54–1.70)	0.89
38–48	0.61 (0.29–1.30)	0.20	0.47 (0.18–1.23)	0.13
Smoking	0.97 (0.62–1.51)	0.90	1.06 (0.62–1.81)	0.84
Magnesium sulphate	1.85 (1.28–2.7)	<0.01	1.44 (0.90–2.30)	0.13
Antenatal corticosteroids	0.50 (0.35–0.72)	< 0.01	0.41 (0.26–0.64)	<0.01
Twins	0.94 (0.65–1.35)	0.73	1.09 (0.70–1.70)	0.71
Male	1.26 (0.89–1.79)	0.20	1.11 (0.72–1.71)	0.64
Social security benefit for low incomes	0.73 (0.43–1.25)	0.25	0.73 (0.39–1.40)	0.35
High socio-economic status	0.58 (0.35–0.96)	0.04	0.93 (0.50–1.70)	0.80

(BMI Body Mass Index, OR Odd Ratio, CI Confidence Interval)

## Discussion

### Main findings

The aim of this study was to study the relationship between maternal BMI and neonatal outcome and neurological outcome at 2 years for preterm infants born before 34 weeks of gestation. We found no difference in neonatal outcome and global neurodevelopment at 2 years between preterm infants born of overweight, obese, or normal BMI mothers.

### Interpretation

#### Neurodevelopmental outcome

In our study, adverse pregnancy outcomes, such as miscarriages, preeclampsia, gestational diabetes, IUGR, were increased in obese women as described in the literature [5]. These poor outcomes may be partially due to inflammation created by adipose tissue in obese mothers. Indeed, maternal obesity leads to a lipotoxic placental environment that is associated with decreased regulators of angiogenesis and increased markers of inflammation and oxidative stress with placental dysfunction and impaired foetal growth [13, 25, 26]. This inflammatory state may be an independent risk factor for the development of cerebral palsy in children of obese mothers because the inflammatory environment may increase susceptibility to hypoxic-ischemic injury. The excess cerebral palsy risk associated with morbid obesity was mainly observed on studies focused on term infants [27–31]. Among Swedish women with singleton children, maternal obesity and being overweight were significantly associated with the rate of cerebral palsy but the association was limited to children born at full term and was partly mediated through asphyxia-related neonatal complications [14]. Few studies examined the link between maternal BMI and the neurodevelopment of preterm infants. These exclusively involved extreme preterm infants born before 28 weeks of gestation and before 30 weeks of gestation [15]. Compared to infants born from mothers with normal BMIs, extreme preterm infants from obese mothers were more likely to have Bayley Scale scores more than 3 standard deviations below the reference mean at the age of 2; maternal obesity was also associated with a positive screening for autism and a lower composite language score. We hypothesise that the excess cerebral palsy risk associated with morbid obesity was observed only for moderate preterm or term infants, in preterm infants the majority of risk is likely attributed to extreme prematurity rather than maternal characteristics [13]. Other prematurity-related factors might be more relevant for neurodevelopmental outcome than pre-pregnancy mother BMI.

In our study, antenatal corticosteroids and high socio-economic status were both independently associated with a decrease in non-optimal neurodevelopmental outcomes at 2 years as described in the literature [32–33]. Male status and gestational age were also an independent factor of non-optimal neurodevelopmental outcome at 2 years in our study as described in the literature [34;40].

#### Neonatal outcomes

Regarding preterm infants, few studies have focused on the association between maternal BMI and neonatal complications. Maternal obesity increases the risk of macrosomia mainly in the third trimester and also an increase in the risk of placental diseases such as preeclampsia and/or IUGR which can lead to a preterm birth. The results from birth weight are quite heterogeneous according to gestational age. Maternal obesity was not associated independently with adverse neonatal outcomes in other studies [35–38].

In our study, antenatal corticosteroids and gestational age were associated with a decrease in non-optimal neonatal outcomes as described in the literature [39,40].

### Strengths and limitations

One limit of our study is that it is a cohort study with missing prenatal data. Indeed, weight and height before pregnancy were self-reported by mothers at their first prenatal visit between 12 and 14 gestational weeks, and retrospectively collected from the medical records for this study in order to calculate BMI. Self-reported weight and height during pregnancy could also be a recall bias in our study. Nevertheless, perinatal, neonatal or follow up data were prospectively collected based on our regional network (LIFT network) and with a follow-up rate of 81% at 2 years of corrected age. Another limitation is that our study is a single-centre study. Given the sample size of our study, the minimum crude ORs detectable for a statistical power of 0.80, were 1.70 and 1.90 for the relationship between maternal obesity and being overweight and neonatal outcome, respectively, and 1.98 and 2.14 for the relationship between maternal obesity and being overweight and neurodevelopmental outcome at 2 years, respectively. Below these values, we cannot conclude whether the results were really not significant (absence of effect of maternal BMI) or not significant due to a lack of statistical power. However, because all the ORs found in our study were <1, it is unlikely that we failed to show a relationship due to a lack of statistical power. Finally, the 2-year neurodevelopmental outcome was assessed by a combination of tests and questionnaires not widely used: a standardised clinical examination, a psychometric test mainly used in France (the revised Brunet-Lézine test) and a parental questionnaire (ASQ). More standardised tests and/or assessments at an older age may yield other results.

## Conclusion

In this large prospective cohort of preterm infants born before 34 weeks of gestation and as measured in our study for neonatal and neurodevelopmental outcome, we found no relationship between maternal BMI and neurodevelopmental outcome at 2 years and no relationship between maternal BMI and neonatal outcome. Other prematurity-related factors may be more relevant for neurodevelopmental outcome than pre-pregnancy mother BMI.

## Supporting information

S1 Table Maternal and neonatal characteristics according to neurodevelopmental assessment at 2 years of corrected age. Number (%) *(BMI Body Mass Index; IUGR Intra-Uterine Growth Restriction*, *SD Standard Deviation) Z-score*: *Birth weights were expressed in relation to gestational age as z-scores for standard deviations (SD) from Olsen growth curves*.
(DOCX)
