# Supplementary material for: Neonatal and neurodevelopmental outcomes in preterm infants according to maternal body mass index: A prospective cohort study
Source: PLoS One. 2019 Dec 5;14(12):e0225027. doi: 10.1371/journal.pone.0225027 (PMC6894768; doi:10.1371/journal.pone.0225027)
Supplement: S1 Table — Number (%) (BMI Body Mass Index; IUGR Intra-Uterine Growth Restriction, SD Standard Deviation) Z-score: Birth weights were expressed in relation to gestational age as z-scores for standard deviations (SD) from Olsen growth curves. (DOCX) [file pone.0225027.s001.docx]

|  | **Infants not assessed at 2 years**  **n= 323** | **Infants assessed at 2 years**  **n=520** | **p value** |
| --- | --- | --- | --- |
| Maternal BMI (kg/m²)  18-25  25-30  >30 | 136 (68,3)  37 (18,6)  26 (13,1) | 338 (65)  111 (21,3)  71 (13,7) | 0,667 |
| Maternal age (years)  16-24  25-37  38-48 | 60 (20,4)  205 (69,7)  29 (9,9) | 84 (16,2)  382 (73,5)  54 (10,4) | <0,001 |
| Primiparous | 100 (31,4) | 186 (35,8) | 0,228 |
| Social security benefits for individuals with low incomes | 32 (9,9) | 77 (14,8) | 0,052 |
| High socio-economic level | 33 (10,2) | 102 (19,6) | <0,001 |
| Smoking during pregnancy | 80 (29,6) | 88 (16,9) | <0,001 |
| Malformative pathology | 8 (2,5) | 17 (3,3) | 0,658 |
| Pregnancy induced hypertension | 12 (3,7) | 12 (2,3) | 0,326 |
| Pre eclampsia / HELLP syndrome | 53 (16,4) | 121 (23,3) | 0,021 |
| Pre-gestational diabetes | 4 (1,2) | 4 (0,8) | 0,491 |
| Gestational diabetes | 28 (8,7) | 49 (9,4) | 0,805 |
| Chorioamnionitis | 17 (5,3) | 14 (2,7) | 0,08 |
| Intra uterine growth restriction | 39 (12,1) | 58 (11,2) | 0,755 |
| Premature rupture of membranes >24 hours | 54 (16,8) | 82 (15,8) | 0,774 |
| Preterm labour (before 34 weeks of gestation age) | 168 (52,2) | 241 (46,3) | 0,116 |
| Medically assisted procreation | 44 (13,7) | 110 (21,2) | 0,008 |
| Caesaren | 201 (62,2) | 327 (62,9) | 0,906 |
| Magnesium sulfate | 53 (17,4) | 145 (27,9) | <0,001 |
| Prenatal steroids (1 injection or more) | 252 (80) | 465 (89,4) | <0,001 |
| Gestational age (weeks)  32-34  28-31  24-27 | 152 (47,2)  110 (34,2)  60 (18,6) | 208 (40)  244 (46,9)  68 (13,1) | <0,001 |
| Gender male | 175 (54,3) | 282 (54,2) | 1 |
| Twin status | 96 (29,8) | 184 (35,4) | 0,111 |
| Birth weight Z-score  < -1 SD  -1 – 0 SD  0 - +1 SD  >+1 SD | 62 (19,7)  107 (34)  106 (33,7)  40 (12,7) | 116 (22,4)  180 (34,7)  165 (31,9)  57 (11) | 0,709 |
| Neonatal morbidity | 87 (26,9) | 125 (24) | 0,389 |

**S1 Table: Maternal and neonatal characteristics according to neurodevelopmental assessment at 2 years of corrected age:** number (%)

*(BMI Body Mass Index; IUGR Intra-Uterine Growth Restriction, SD Standard Deviation)*

*Z-score: Birth weights were expressed in relation to gestational age as z-scores for standard deviations (SD) from Olsen growth curves*
